# Prospective prescription review system correlated with more rational PPI medication use, better clinical outcomes and reduced PPI costs: experience from a retrospective cohort study

**DOI:** 10.1186/s12913-023-09931-5

**Published:** 2023-09-20

**Authors:** Xiucong Fan, Danxia Chen, Siwei Bao, Xiaohui Dong, Fang Fang, Rong Bai, Yuyi Zhang, Xiaogang Zhang, Weijun Tang, Yabin Ma, Xiaobo Zhai

**Affiliations:** Department of Pharmacy, Shanghai East Hospital, Tongji University School of Medicine, Shanghai, 200123 China

**Keywords:** Prospective prescription review, Proton pump inhibitors, Clinical pharmacists, Propensity score matching, Rational drug use

## Abstract

**Introduction:**

Proton pump inhibitor (PPI) abuse poses an overwhelming threat to the allocation of medical resources and places a heavy burden on global medical expenses. In this study, we put forward our prospective prescription review system and evaluated the effects of this system on clinical outcomes, rational medication use and costs related to PPIs.

**Methods:**

A retrospective cohort study was conducted in which the included patients were divided into a preintervention group (2019.10–2020.09) and a postintervention group (2020.10–2021.09). To reduce the bias of patients’ baseline characteristics, the propensity score matching (PSM) method was employed. The primary endpoints were the incidence of stress ulcers (SUs), the improvement and cure rates of gastrointestinal haemorrhage, the defined daily dose (DDD), the drug utilization index (DUI) and the DDD/100 patient-days. The secondary endpoints included the types of unreasonable medication orders for PPIs, the PPI utilization rate and PPI costs.

**Results:**

A total of 53,870 patients were included to evaluate the secondary endpoints, and 46,922 patients were paired by PSM and assessed to evaluate the primary endpoints. The number of PPIs used and PPI costs were significantly lower in the postintervention group than in the preintervention group (P < 0.001). The rationality evaluation results showed that the frequency of PPI use and the number of drug interactions were significantly higher in the preintervention group than in the postintervention group (P < 0.01). The proportion of patients taking oral PPIs was significantly increased in the postintervention group (29.30% vs. 34.56%, p < 0.01). For the utilization of PPIs both for prevention and treatment, the DUI and DDD/100 patient-days were substantially decreased in the postintervention group (P < 0.001 and P < 0.05, respectively). The incidence of SUs in the postintervention group was 44.95%, and that in the preintervention group was 51.93% (p < 0.05).

**Conclusion:**

The implementation of the prospective prescription review system on rational PPI use correlated with reduced PPI costs, more rational PPI medication use and better clinical outcomes, and this system is worthy of long-term implementation for further improvement of rational drug use.

## Introduction

Critically ill patients are at high risk for experiencing stress ulcers (SUs). When using a positive faecal occult blood test or unexplained haemoglobin decline as the diagnostic criteria, the incidence of SUs in critically ill patients ranges from 15 to 50% [1]. To prevent the occurrence of SUs, proton pump inhibitors (PPIs) are considered effective and rank as the most commonly prescribed drugs worldwide [2]. However, with the surge in PPI expenditures, the irrational use of PPIs without evidence has increased rapidly [3], resulting in unreasonable financial expenditures. Forgacs I et al. [4] indicated that almost £2 billion are being spent on PPIs with unnecessary medication purposes each year worldwide. In addition to the economic waste of PPIs, adverse effects of long-term use and potential disadvantages in health issues have been identified and attracted the attention of researchers. The adverse effects of long-term use included increased risks of infections (lungs and gastrointestinal tract), bone fractures, liver and kidney damage, as well as a decrease in absorption of vitamins and minerals from the intestine [5–9]. In terms of potential harms, drug-drug interactions (DDIs) may occur when PPIs are added to polytherapy, which could result in unpredictable outcomes and threaten patients’ health [10].

In recent years, various measures have been taken to improve the abuse of PPIs globally. Clinical pharmacists, as the initiators of rational drug use, have been actively involved in this work and have achieved satisfactory results. Khalili H et al. [11] reported that clinical pharmacists introduced a treatment guideline for stress ulcer prophylaxis (SUP) based on a warranted evidenced protocol and assisted physicians in prescribing acid-suppressive therapy (AST) for SUP, which obtained reductions in the overall use of AST and the use of AST for patients without reasonable indications. Masood U et al. [12] showed that their clinical pharmacist team reviewed SUP patients during medical rounds, made appropriate changes according to the guidelines and educated residents and fellows on the implemented initiative of SUP, which caused a substantial reduction in the costs related to inappropriate SUP use. Moreover, Mitchell S et al. [13] described that their team owned prescriptive authority on decreasing inappropriate stress ulcer prophylaxis rates instead of recommendations, which could be more time-efficient and cost-saving. As in China, the “Consensus Review for SUP and Treatment” and “Prevention and Treatment of Stress Related Mucosal Disease” were released in 2015 and to some extent promoted the rational drug use of PPIs and the prevention of SUs [14]. Under the guidance, Chinese clinical pharmacists have been taking interventions to control PPI overuse on the premise of reducing the incidence of SUs in hospitals. The majority of the interventions are focused on daily rounds with physicians and educative group activities about the rational use of PPIs [15, 16]. Some pharmacists have explored classic management methods for improving rational PPI use. Yun H et al. [17] studied the application of the plan-do-check-act (PDCA) method in promoting rational prophylactic injectable proton pump inhibitor use (PIPU), while clinical pharmacists led a guidance team in providing pharmaceutical care.

In China, the prospective prescription review system is a real-time prescription monitoring system that requires clinical pharmacists to set rules for intercepting prescriptions with drug-related problems (DRPs) according to clinical guidelines, medical insurance policy, drug descriptions, etc. Since the National Health Commission issued a prescription review of medical institutions in July 2018, approximately 42.7% of hospitals have successively carried out a prospective prescription review system and achieved remarkable results [18]. Xie H et al. [19] reported that the introduction of the prescription review system was associated with safer prescribing of analgesics. However, to date, few studies have examined prospective prescription review systems regarding the promotion of rational PPI use.

Therefore, the clinical pharmacists in our hospital developed a prospective prescription review system correlating PPI prescriptions to the fasting state and all the related diagnoses of stressors. This system attempts to improve clinical outcomes associated with gastrointestinal bleeding while controlling unreasonable PPI use and reducing unnecessary PPI expenses.

## Methods

### Study setting

This was a retrospective preintervention/postintervention study conducted in Tongji University affiliated East Hospital. Tongji University affiliated East Hospital is a tertiary teaching hospital with 1800 beds in Shanghai. This hospital of two districts owns 9 ICUs and a wide range of general wards, including the Department of Cardiology, Department of Obstetrics and Gynecology, Department of Oncology, etc. In October 2018, the clinical pharmacists in the Department of Pharmacy launched a prescription review system, which provided a real-time prescription review for all outpatients and inpatients. The study protocol was approved by the Tongji University affiliated East Hospital Review Board (2020-092). The need for written informed consent was waived by the Research Ethics Committee of Tongji University affiliated East Hospital due to the retrospective nature of the study. All methods were conducted following relevant guidelines and regulations.

### Risk assessment and prevention system of stress ulcers

**Fig. 1:**
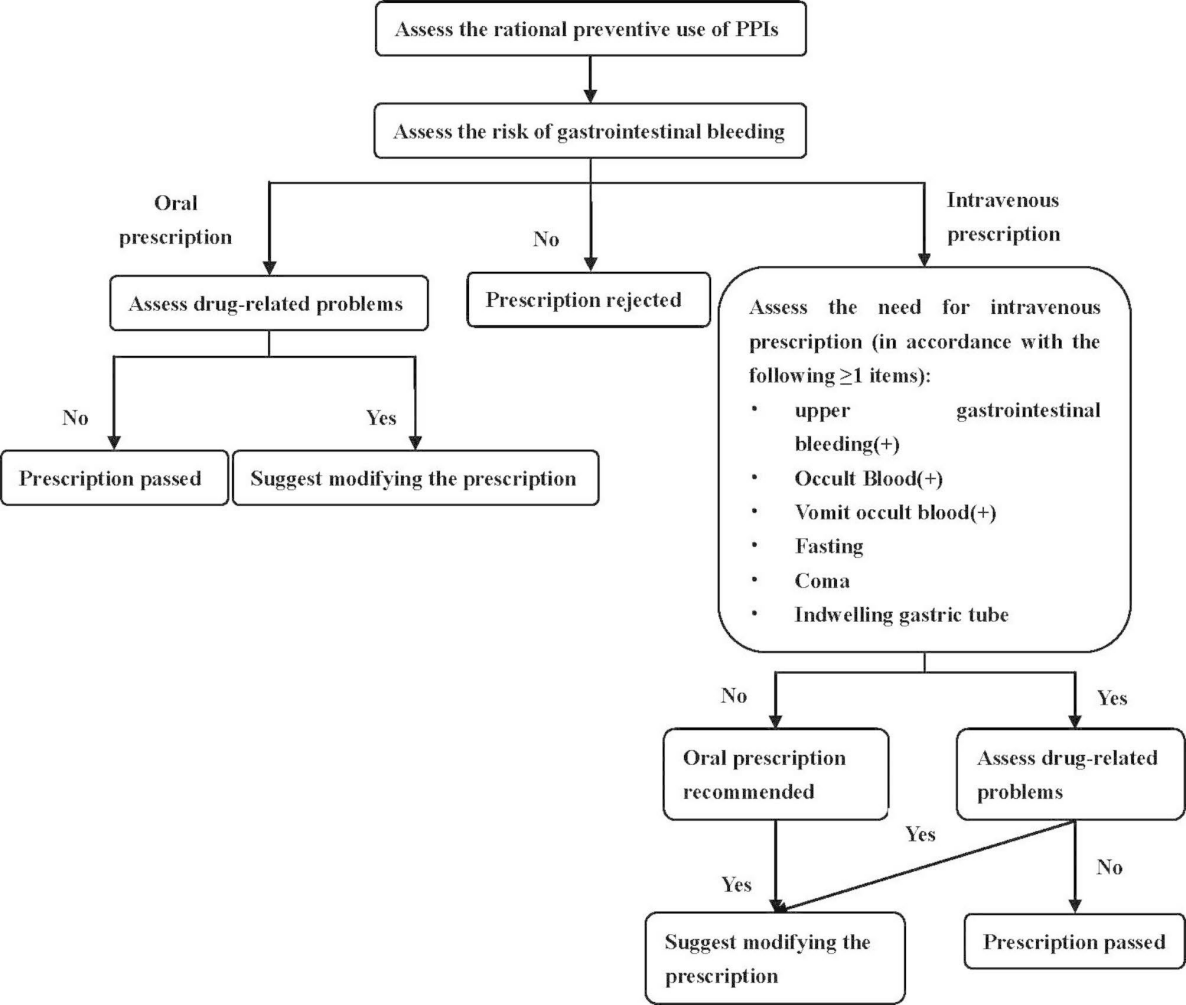
Flow chart of the prospective prescription review system for rational PPI drug use

The establishment of risk assessment and prevention of SUs was based on a prospective prescription audit system, which intends to remind doctors of prescriptions that may lead to DRPs. When there is DRP in the prescription issued by the doctor, the warning message will automatically pop up in the doctor’s work interface. If the doctor does not modify the prescription according to the warning message, he or she can select “request dispensing”. Then, the clinical pharmacist will receive this prescription in his or her work interface and will write recommendations according to the patient’s condition, and the clinical pharmacist will send the recommendations back to the doctor’s work interface for interaction. The doctor can choose to modify the prescription according to the pharmacist’s recommendations or reject the recommendations. On the premise of continuing the workflow above, the risk assessment and prevention system of stress ulcers aims to dynamically monitor and evaluate stressors and risk factors and promote the timely adjustment of the dosage and course of PPI treatment. It realizes the connection between PPIs and fasting state and all the related diagnoses of stressors. The details are as follows: (1) When doctors prescribe PPIs for patients without stressors or risk factors, the system will give timely warnings to intercept the prescriptions. (2) When doctors prescribe oral PPIs for patients at risk of gastrointestinal bleeding (stressor ≥ 1, or risk factors ≥ 2), the system will judge whether there are DRPs, and the warning message will automatically pop up in the doctor’s work interface. (3) When doctors prescribe intravenous PPIs for patients at risk of gastrointestinal bleeding (stressor ≥ 1, or risk factors ≥ 2), based on the process above, whether one more step to determine if intravenous administration is needed should be judged by the system. [Fig. 1].

### Subject

The study patients were divided into two cohorts according to the implementation of the system: a preintervention cohort (2019.10-2020.9) and a postintervention cohort (2020.10-2021.9). We reviewed all the in-hospital prescriptions during this period. Two clinical pharmacists collected medical information on the patients’ demographics (sex, age, inpatient department, purpose of PPI use and length of hospital stay), diagnoses, drug information (drug name, dosage form, usage dates, specification, usage and dosage, and expenditures) and detailed in-hospital expenses from the Electronic Medical Record System (EMR). Data on intravenous PPI review points and irrational PPI evaluation results were collected from the prospective prescription system. Then, the collected data were cross-checked by two senior pharmacists. The inclusion criteria were as follows: (1) Patients with in-hospital prescriptions including PPIs (both the oral and intravenous administration forms); (2) Patients aged ≥ 18 years; and (3) Patients with admission and discharge diagnoses related to Zollinger-Ellison syndrome, peptic ulcer, gastroesophageal reflux, upper gastrointestinal bleeding, *Helicobacter pylori* infection and SUs. The exclusion criteria included the following: (1) Patients who were not discharged during the data extraction period; (2) Patients with incomplete in-hospital data; (3) Patients with histamine-2 receptor antagonist (H2RA) prescriptions during the in-hospital period; and (4) Patients with an admission time less than 48 h.

### Variables and outcomes

Stressors were defined as underlying diseases that induced SUs, which mainly included the following [20]: (1) Mechanical ventilation duration > 48 h; (2) Coagulation mechanism disorder (an internal normalised ratio (INR) > 1.5, a platelet level < 50 × 10^9^/L or a partial prothrombin time > 2); (3) History of peptic ulcer or upper gastrointestinal bleeding within 1 year; (4) Severe head injury and cervical spinal cord injury, with a Glasgow Coma Score ≤ 10 points(or cannot execute simple commands); (5) Severe burns (burn area＞30%); (6) Severe trauma and multiple injuries; (7) Various difficult and complicated operations (operation time > 3 h, operation grade ≥ 3); (8) Kidney dysfunction or renal replacement therapy; (9) Acute liver dysfunction or chronic liver disease; (10) Acute respiratory distress syndrome (ARDS); 11) Shock or persistent low blood pressure (persistent low blood pressure duration＞30 min; shock refers to a systolic blood pressure < 90 mmHg or lower than a basal blood pressure > 40 mmHg); 12) Sepsis; 13) Cardiovascular accident; and 14) Severe psychological stress, due to mental trauma or excessive stress.

The risk factors for Sus included [20, 21]: (1) An ICU hospital stay > 1 week; (2) A positive faecal occult blood duration > 3 days; (3) High-dose corticosteroid administration (250 mg/day of steroids or equivalent hydrocortisone); and (4) Nonsteroidal anti-inflammatory drug (NSAID) administration.

SUs were defined as no diagnosis related to gastrointestinal bleeding that was present in the admission diagnosis, but a diagnosis related to gastrointestinal bleeding or stress ulcer was demonstrated in the discharge diagnosis. SUs occurred in patients who were previously given preventive PPIs based on risk factors and stressors, related medical history, etc. PPI medications then replaced preventive PPI medications for treatment once upper gastrointestinal haemorrhage symptoms, signs and laboratory examination abnormalities appeared.

The defined daily dose (DDD) of PPIs was defined as “the average maintenance dose of a drug when used for its major indication in adults” and was calculated according to the recommendations in the WHO ATC/DDD Index 2019, the “Pharmacopeia of the People’s Republic of China-Clinical Medication Instructions” (2015 edition), the “Chinese Pharmacist and Physician Clinical Medication Guide”, etc.

The DDDs was calculated as follows: total PPI consumption/DDD. The higher the DDDs value was, the higher the frequency of use of the drug.

The drug utilization index (DUI) was calculated as follows: the sum of the DDDs of all PPIs for patients in each group/the total number of days the medication was used by the patients. The DUI can be used to evaluate the rationality of clinical medication use. A DUI greater than 1.0 means that the doctor’s daily dose is greater than the DDD, demonstrating unreasonable medication use.

The DDDs/100 patient-days was calculated as follows: the average DDDs of PPIs consumed per 100 patient-beds per day.

The SU incidence (%) was calculated as follows: the number of patients with SUs/the number of all patients with preventive PPI use ×100%.

Improvement and cure of gastrointestinal haemorrhage refer to the evaluation of improvement or cure added in the diagnosis related to gastrointestinal bleeding in the discharge diagnosis. Evaluation of the improvement rate and cure rate of gastrointestinal haemorrhage was performed for the patients who experienced PPI treatment medications.

The improvement and cure rates of gastrointestinal haemorrhage (%) was calculated as follows: the number of patients with improved or cured gastrointestinal haemorrhage/the number of all patients with PPI use ×100%.

PPI costs were calculated as follows: the number of patients with PPI drug costs in each group/the total number of patients in each group.

The Charlson Comorbidity Index (CCI) [22] is a weighted index that considers the number and seriousness of comorbid diseases according to a point system based on 19 comorbidities. Every comorbidity was assigned a weight of 1, 2, 3 or 6, and the CCI score was calculated by summing the weight of comorbidities. A CCI score of 0 indicated no comorbidities; a CCI score of 1 indicated one comorbidity; a CCI score of 2 indicated two comorbidities weighted as 1 or one comorbidity weighted as 2; a CCI score of 3 indicated three comorbidities weighted as 1 or one comorbidity weighted as 1 and one comorbidity weighted as 2; and a CCI score ≥ 4 indicated an estimated 10-year survival rate of less than 53%.

The primary endpoints were grouped as the incidence of SUs, the improvement rate and cure rate of gastrointestinal haemorrhage, the DDD, the DUI and the DDD/100 patient-days. The secondary endpoints included types of unreasonable medication orders for PPIs, the PPI utilization rate and PPI costs.

### Statistical analysis

In this study, statistical analysis was conducted using SPSS 25.0 software (SPSS Inc., Chicago, IL, USA) for Windows. We used propensity score matching (PSM) to reduce the cofounding bias when comparing the clinical outcomes between the groups. To apply PSM, logistic regression was employed to calculate the propensity score values, which included age, sex, in-hospital departments, the CCI score and the PPI medication purpose. Nearest neighbour matching (1:1) was used as the matching method to calculate the standardized differences before and after PSM. When the variable fell between 0.0 ± 0.001, the matching then was stopped as a sign of reaching equilibrium. Continuous variables are presented as medians along with standard deviations (SDs) and were tested by grouped t tests. Other quantitative data are presented as the upper quartile (Q1) and lower quartile (Q3). Q2 is represented as the median. The number of patients (percent) was described categorically, and frequency comparison was analysed by the chi-square test or Wilcoxon rank sum test. P values equal to or less than 0.05 were considered statistically significant.

## Results

A flow chart of the prospective prescription review is shown in Fig. 1. This figure shows how the intervention was performed on the rational use check of PPIs.

Table 1 presents the comparisons of PPI use and PPI costs. The number of PPIs used and PPI costs were significantly lower in the postintervention group than in the preintervention group (P < 0.001).

**Table 1:** Comparisons of PPI use and PPI costs

	preintervention group (n = 24,560)	postintervention group (n = 29,310)	P value
PPI used(%)	11,774 (47.94)	12,439 (42.44)	< 0.001
PPI not used(%)	12,786 (52.06)	16,961 (57.56)	
PPI costs (in¥, Q1, Q3)	517.75 (66.93,519.41)	441.84 (29.55,407.92)	< 0.001

**Fig. 2:**
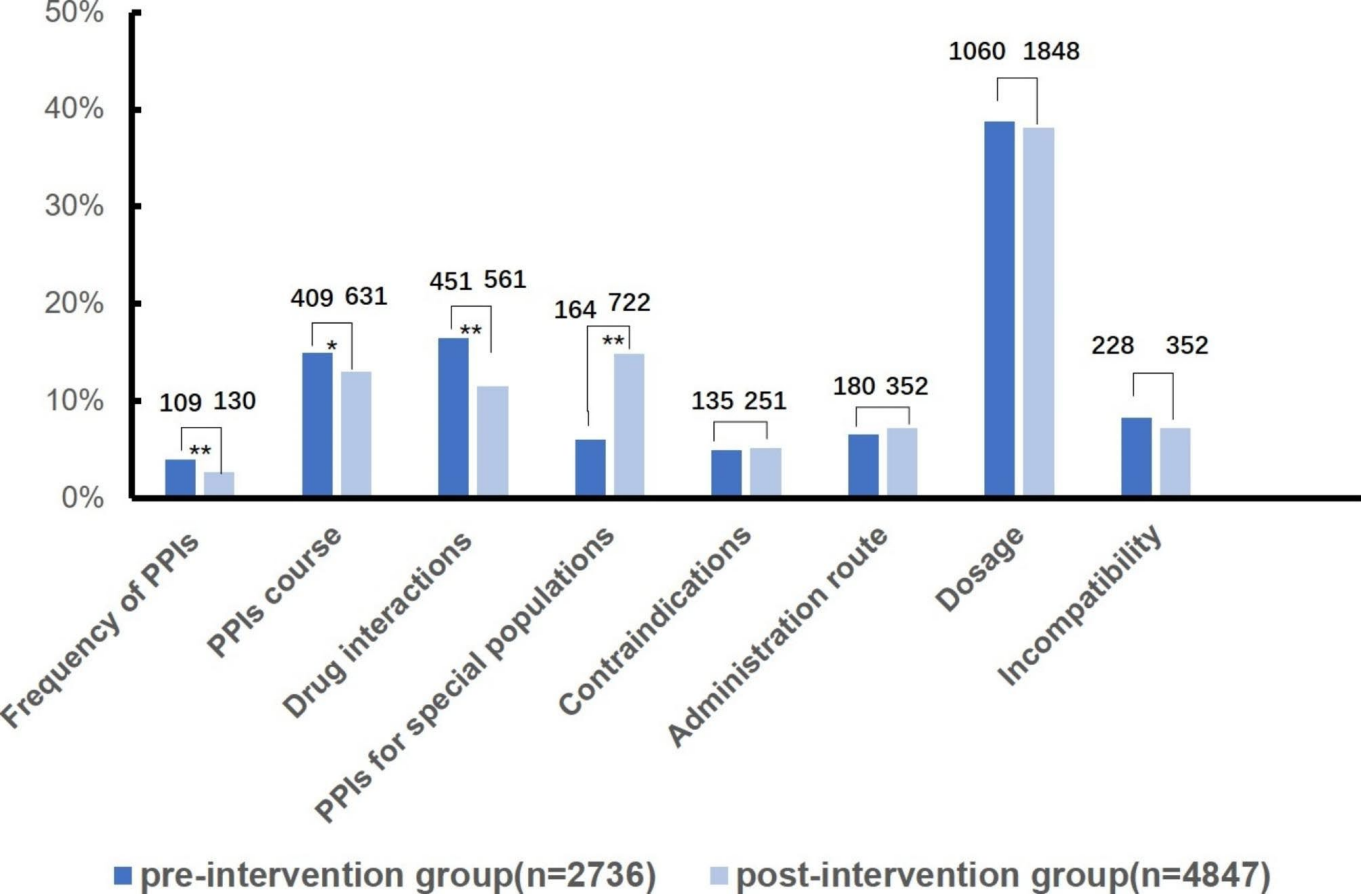
The distribution and comparison of the irrational evaluation results of PPI use between the preintervention group and the postintervention group (*P < 0.05, **P < 0.01)

According to the data shown in Fig. 2, the number of unreasonable medical orders was 2736 and 4847 in the pre- and postintervention groups, respectively. The rationality evaluation results were categorized as follows: frequency of PPIs, PPIs course, drug interactions, PPIs for special populations, contraindications, administration route, dosage and incompatibility. The most common problem was the dosage, with 1060 (38.74%) and 1848 (38.13%) patients in the pre- and postintervention groups, respectively. The composition ratios of the frequency of PPIs and drug interactions were significantly higher in the preintervention group than in the postintervention group (P < 0.01). The PPI course in the preintervention group had a higher composition ratio than that in the postintervention group (P < 0.05). Of note, the composition ratio of PPIs for the special populations in the preintervention group was significantly lower than that in the postintervention group (P < 0.01).

**Fig. 3:**
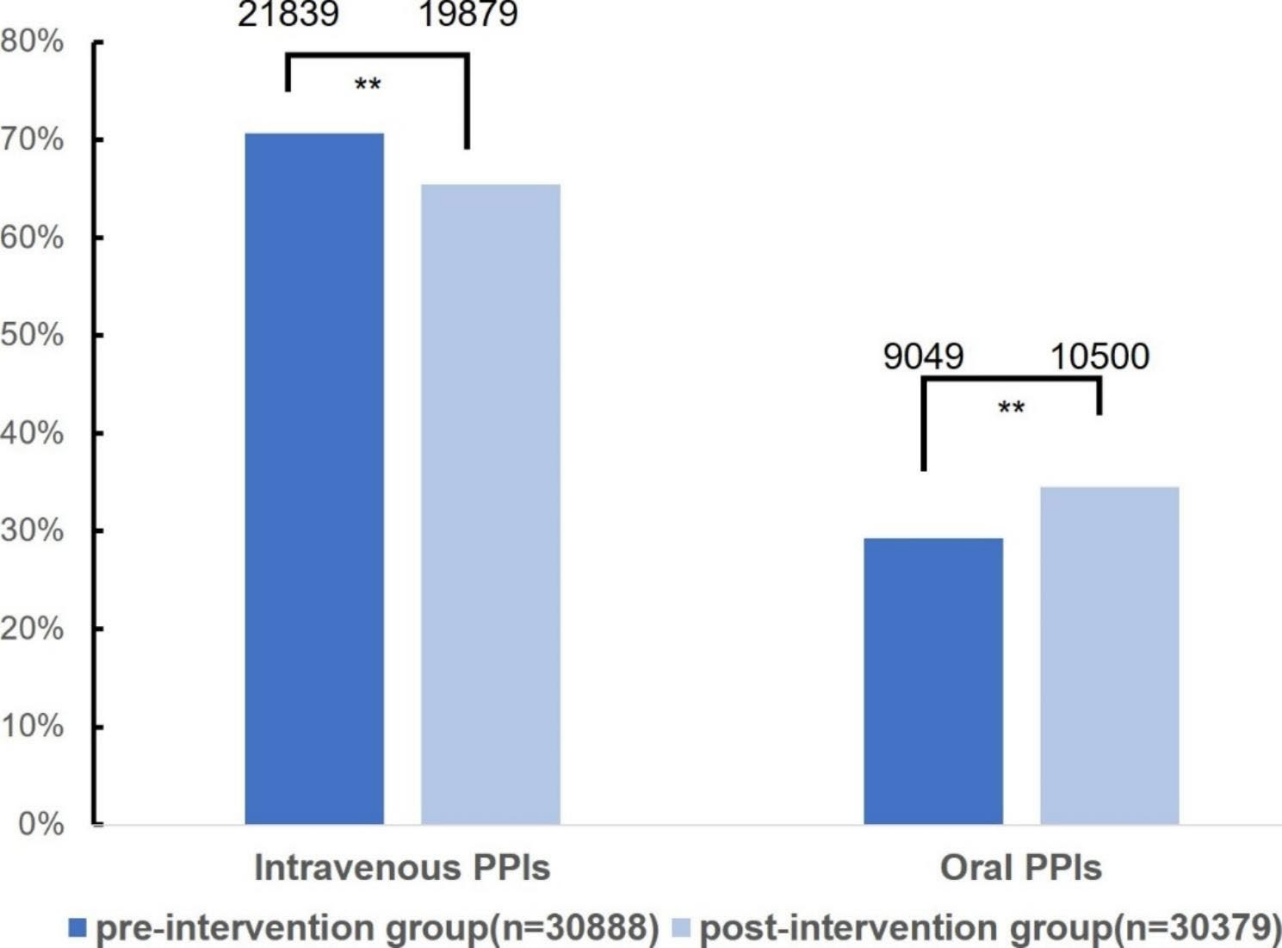
The comparison of the medical orders for intravenous PPIs and oral PPIs in the preintervention group and postintervention group (**P < 0.01)

As shown in Fig. 3, the number of medication orders in the pre- and postintervention groups was 30,888 and 30,379, respectively. The proportion of oral PPIs increased significantly after intervention (29.30% vs. 34.56%, p < 0.01), and there was a significant difference in unreasonable medical orders (8.86% vs. 15.96%, p < 0.01) between the two groups.

**Fig. 4:**
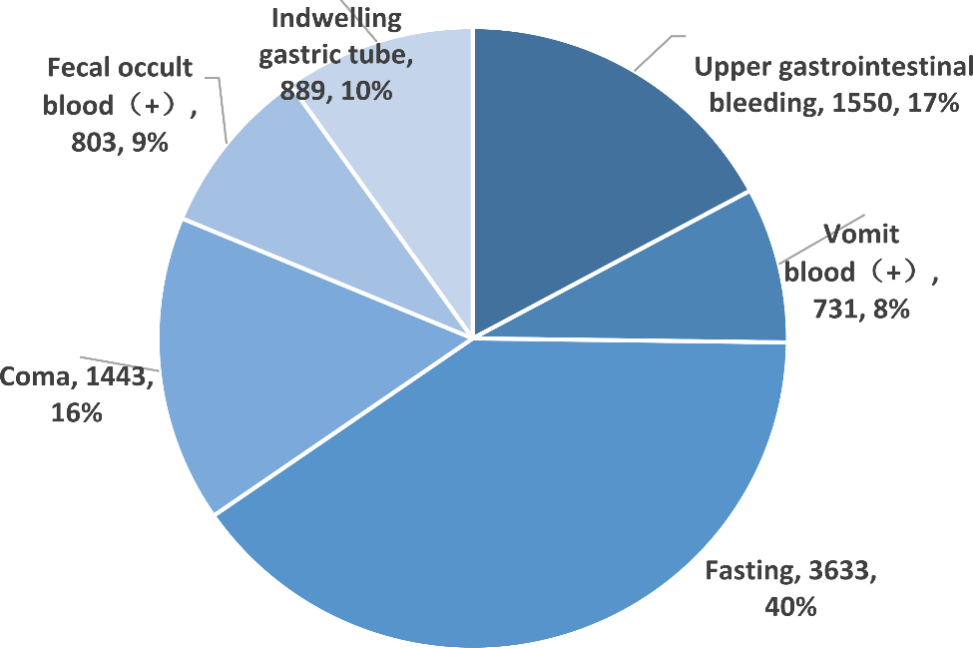
The review points of intravenous PPIs that have to be selected before an intravenous PPI medical order is prescribed

The review points of intravenous PPIs were presented as follows: upper gastrointestinal bleeding, vomiting blood (+), fasting, coma, faecal occult blood (+) and indwelling gastric tube. When doctors prescribed intravenous PPIs, the most common review point they chose was fasting (3633, 40.15%), followed by upper gastrointestinal bleeding (1550, 17.19%) and coma (1443, 15.95%) (Fig. 4).

The characteristics of the patients included in the analysis are shown in Table 2. Since the characteristics of age, sex, department, and CCI score were not comparable between the two groups, PSM was applied to make them comparable. After the adjustment for the demographic variables by PSM analysis, there were no significant differences in age, sex, department, CCI or purpose of PPI use between the two groups.

**Table 2:** Baseline characteristics before and after PSM in the pre- and postintervention groups

Demographic Characteristics		Before PSM				After PSM		
		Preintervention group (n, %)	Post-intervention group(n,%)	P value		Preintervention group (n, %)	Post-intervention group(n,%)	P value
Sex, male		13,811(56.23)	16,407(55.98)	0.551		13,142(56.02)	13,142(56.01)	1
Age (years) mean ± SD		61.6 ± 16.3	62.2 ± 16.0	< 0.01		61.8 ± 16.2	61.5 ± 16.1	0.274
＜65 years		13,633(55.51)	15,528(52.98)	< 0.01		12,170(51.87)	12,098(51.57)	0.506
＞65 years		10,927(44.49)	13,782(47.02)			11,291(48.13)	11,363(48.43)	
Department	ICU	1222(4.97)	1481(5.05)	0.682		1170(4.99)	1098(4.68)	0.121
	Surgical Department	13,450(54.76)	14,113(48.15)	< 0.01		12,771(54.44)	12,771(54.44)	1
	Internal Medicine Department	9888(40.26)	13,716(46.80)	< 0.01		9520(40.58)	9592(40.88)	0.499
CCI	0	10,362(42.19)	10,581(36.10)	< 0.01		9689(41.30)	9712(41.40)	0.821
	1	3981(16.21)	4207(14.35)	< 0.01		3389(14.44)	3415(14.56)	0.733
	2	4984(20.29)	4995(17.04)	< 0.01		4701(20.04)	4602(19.62)	0.252
	3	2271(9.25)	2947(10.05)	0.002		2235(9.53)	2209(9.42)	0.682
	≥ 4	2962(12.06)	6580(22.45)	< 0.01		3447(14.69)	3523(15.02)	0.324
Purpose of PPI use	Treatment	439(1.79)	472(1.61)	0.112		389(1.66)	416(1.77)	0.373
	Prevention	24,121(98.21)	28,838(98.39)			23,072(98.34)	23,045(98.23)	

The purpose of PPI use was divided into prevention and treatment. For the utilization of PPIs for prevention, the total DDDs (231,765 vs. 202,541) and the DDDs/100 patient-days (110.71 ± 70.02 vs. 100.71 ± 69.68) were substantially decreased in the postintervention group (P < 0.001). Moreover, the DUIs between the two groups were 1.52 and 1.4, respectively. The number of days stayed in the hospital was significantly different between the two groups. For the utilization of PPIs for treatment, DUI and DDDs/100 patient-days were significantly different between the pre- and postintervention groups. The incidence of SUs in the postintervention group was 44.95%, and that in the preintervention group was 51.93% (P < 0.05). The improvement rate and cure rate of gastrointestinal haemorrhage in the two groups were not significantly different (Table 3).

**Table 3:** Comparisons of the patients’ clinical outcomes and evaluation index of rational PPI use

	PPIs for prevention					PPIs for treatment			
	Preintervention group (n = 23,072)		Post-intervention group(n = 23,045)	P value		Preintervention group(n = 389)		Post-intervention group(n = 416)	P value
Improvement rate and cure rate of gastrointestinal haemorrhage (n, %)						299(76.86)		320(76.92)	0.504
Incidence of SUs (n, %)						202(51.93)		187(44.95)	0.048
Numbers of days in the hospital, median (Q1, Q3)	7(4,12)		6(3,12)	< 0.001		15(8,19)		16(7,19)	0.45
DDDs	231,765		202,541			9814		9805	
DUI, mean ± SD	1.52 ± 0.59		1.4 ± 0.57	< 0.001		2.04 ± 0.79		1.88 ± 0.79	0.01
DDDs/100 patient-days, mean ± SD	110.71 ± 70.02		100.71 ± 69.68	< 0.001		186.25 ± 106.74		172.89 ± 100.81	0.02

## Discussion

Previous studies have shown that clinical pharmacists have achieved satisfactory results in ICUs, surgical wards and medical wards regarding the interventions of reviewing indications for SUP, educating residents, and making ward rounds [12, 23−24]. The uniqueness of this study is that unlike some studies requiring that clinical pharmacists review prescriptions [25, 26], the tasks for clinical pharmacists were to set reasonable interception rules and wait for feedback from doctors when alerts occurred, which is a more efficient and cost-effective method. The findings in this study show that in the postintervention group, the total PPI utilization rate (PPIs used) and PPI costs decreased significantly compared to those in the preintervention group. The total PPI utilization rate in this study was lower than some of the previous studies domestically and worldwide [16, 27]. This means that reminding doctors of the utilization rules with this system has achieved the initial goal. In addition, the clinical pharmacists in our hospital have given PPI recommendations in clinical pharmacists’ consultations, which to some extent promoted the rational use of PPIs [28]. The findings demonstrated a significant reduction in PPI costs, which is consistent with what Chen QY et al. reported [29]. In October 2019, the government officially implemented the “4 + 7” centralized procurement policy and the price of drugs was greatly adjusted; Shanghai was among the 11 pilot cities. Chinese researchers found that the “4 + 7” policy had positive effects in promoting the substitution of generic drugs for original drugs, reducing drug prices and promoting rational drug use [30, 31]. For the change in PPI costs shown in Table 1, although PPI prices decreased several times during the study period, based on the consumption of drugs, we observed that there was a significant difference in the DDDs between the preintervention group and postintervention group (241,579 vs. 212,346, p < 0.001). The significant decrease in consumption suggested that even though the PPI prices remained stable during the study period, the PPI costs could be significantly reduced after the intervention. This indirectly indicated the significance of the system in reducing PPI costs. Furthermore, we do not need to take cost measurement into consideration in this study. Because clinical pharmacists do not have to spend much time reviewing prescriptions, salaries related to costs were partly saved, unlike the method presented in Bao ZW et al. [25].

We achieved the increased utilization of oral PPIs, instead of intravenous PPIs in this study. As indicated in a previous study [32] and expert consensus [33], for SUP, intravenous PPIs can be selected only when patients are unable to eat, which emphasizes the recommended use of oral PPIs. To improve the current status that includes a high utilization rate of intravenous PPIs and physicians’ lack of realization of indications for intravenous PPIs, we used this system to double check the appropriateness of intravenous PPIs (Fig. 1). Before an intravenous PPI medical order is prescribed, the reasons why intravenous PPIs rather than oral PPIs should be used must be selected (Fig. 4). All the interventions led to a significant reduction in utilization. For the intervention on the various types of irrational drugs, the setting of the rules was more detailed, which means that a prescription could be issued only after the rules were met. For example, one of the rules is that the routine treatment course of intravenously administered PPIs should be less than 7 days. If the treatment course exceeds this duration, the doctor should make sure that the patient is still fasting in the system. Due to the strict restrictions of the rules, the irrational prescriptions of medication frequency and treatment course and those with DRPs were significantly reduced. It is worth noting that the irrational utilization rate of special drug users, including pregnant women, children and people with liver and kidney dysfunction, significantly increased, demonstrating the advantage of this intervention method in intercepting unreasonable prescriptions for these groups over a previous study [13]. We set the rules for this group according to the drug instructions so that more clinical pharmacists’ efforts could influence them.

SUs have the characteristics of a low incidence rate and high mortality. According to the latest finding [34], the bleeding risk of ICU patients receiving SUP can be reduced by approximately 60%. Therefore, prevention is important to reduce the occurrence of SUs, and early application of acid suppressants has been shown to be an effective method. Among the recommended acid suppressants, oral PPIs ranked as the first choice by the guidelines [20, 35]. In this study, the incidence of SUs decreased after the intervention. In addition, the ratio of oral administration was significantly increased, while the ratio of intravenous administration was significantly reduced. The findings of this study showed a much better improvement than the reported research over 20 tertiary hospitals domestically, and this research demonstrated that the use rate of PPIs in hospitalized patients was 57.4% (of which intravenous administration accounted for 82.9%) [36]. Hohl CM et al. [37] reported that a reduced hospital stay could be achieved by pharmacist prescription review. In line with this finding, this study showed a significantly shorter hospital stay in the group that had PPIs for prevention. Furthermore, it is worth noting that the DUI and DDDs/100 patient-days*100 in the postintervention group were significantly lower than those in the preintervention group both for prevention and treatment, demonstrating that the frequency of clinical use and rational drug use was significantly improved. This improvement could be attributed to the implementation of a prospective review system on rational PPI use, with which physicians’ prescriptions could be prospectively reviewed by clinical pharmacists. However, for DUI, the results after the intervention were much higher than the previous findings reported by Zhang Y et al. [16] and Ying J et al. [38], which means multidimensional measures should be implemented to further reduce the DUI. An important issue identified in this study was that the DDDs/100 patient-days after the intervention were 100.71 ± 69.68 and 172.89 ± 100.81 for prevention and treatment, respectively, which were still much higher than the findings of previously reported studies [12, 16]. The reason was that our result covered all the indications of PPIs over the entire inpatient setting, including rational and irrational indications, instead of irrational indications or those limited to only one department.

To the best of our knowledge, this is the first retrospective study to evaluate the improvement of PPI use, patients’ clinical outcomes and medical expenses after implementing a prospective prescription review system in the entire inpatient setting. To reduce bias, a propensity score method was used to match the patients’ conditions. While this study has many methodological strengths, several limitations still exist. First, although this prospective prescription review system reflected the whole picture of how PPIs were used in the real-world inpatient setting, it was a single-centre study. To further study the advancement and advantages of this model, multicentre studies are needed. Second, based on the physicians’ relatively low recognition of the clinical pharmacists’ work, the current acceptance rate of prospective prescription review is only approximately 80%, which means that 20% of physicians choose to sign a guarantee to circumvent the obstruction of the system. Improving the physicians’ recognition is a goal that requires time to complete. A considerable number of physicians believe that clinical pharmacists should improve their professional level [39, 40]. Therefore, continuous improvement of clinical pharmacists’ abilities may be able to reduce these research limitations to some extent. Third, due to some inherent deficiencies in the design of the system, patients who need PPIs but are not prescribed PPIs cannot be included in the unreasonable medication use statistics. To address these issues, more reasonable review rules should be made. Finally, as pointed out in many Chinese studies [41, 42], National Centralized Drug Procurement has been implemented by the Chinese government since 2019. During procurement, the average bid-winning drug price was reduced by 52%. Therefore, the positive effect of the system on total drug costs cannot be accurately evaluated. To counteract this issue, evaluation could be performed after drug prices remain stable.

## Conclusion

In this study, the implementation of the prospective prescription review system on rational PPI use correlated with reduced PPI costs, more rational PPI medications and better clinical outcomes, including a lower incidence rate of SUs and shorter in-hospital days for patients using PPIs for SUP. It is essential that clinical pharmacists and their methods to improve clinical medication use help physicians achieve better medical care quality. To obtain the best effect of this system, clinical pharmacists should develop more rational medication rules to cover more drugs. Furthermore, improving the expertise of pharmacists for higher recognition by physicians is meaningful not only for the system but also for pharmaceutical care.

## Data Availability

The datasets used and/or analysed during the current study available from the corresponding author on reasonable request.

## Abbreviations

ARDS: Acute respiratory distress syndrome
AST: Acid-suppressive therapy
CCI: Charlson Comorbidity Index
DDD: Defined daily dose
DDDs: Total consumption of PPI(g)/DDD
DDDs/100 patient-days: PPI use density
DDIs: Drug-drug interactions
DRPs: Drug-related problems
DUI: Drug utilization index
EMR: Electronic Medical Record System
ICU: Intensive Care Unit
NSAIDs: Nonsteroidal anti-inflammatory drugs
PDCA: Plan-do-check-act
PIPU: Prophylactic injectable proton pump inhibitor use
PPI: Proton pump inhibitor
PSM: Propensity score matching
SUs: Stress ulcers
SUP: Stress ulcer prophylaxis
